# Reducing Asthma Attacks in Children using Exhaled Nitric Oxide as a biomarker to inform treatment strategy: a randomised trial (RAACENO)

**DOI:** 10.1186/s13063-019-3500-7

**Published:** 2019-10-04

**Authors:** S. Turner, S. C. Cotton, C. D. Emele, R. Thomas, S. Fielding, E. A. Gaillard, J. C. de Jongste, H. Morgan, A. R. Neilson, J. Norrie, M. Pijnenburg, D. Price, M. Thomas

**Affiliations:** 1 Child Health, University of Aberdeen, Aberdeen, UK; 2 Centre for Healthcare Randomised Trials, University of Aberdeen, Aberdeen, UK; 3 Medical Statistics Team, Institute of Applied Health Sciences, University of Aberdeen, Aberdeen, UK; 4 Respiratory Sciences, University of Leicester, Leicester, UK; 5 Department of Paediatric Respiratory Medicine and Allergology, Erasmus MC – Sophia Children’s Hospital, Rotterdam, Netherlands; 6 Postgraduate Education Group, Institute of Applied Health Sciences, University of Aberdeen, Aberdeen, UK; 7 Health Economics Research Unit, Institute of Applied Health Sciences, University of Aberdeen, Aberdeen, UK; 8 Observational and Pragmatic Research Institute Pte Ltd, Singapore, Singapore; 9 Centre of Academic Primary Care, University of Aberdeen, Aberdeen, UK; 10 Primary Care and Population Sciences, University of Southampton, Southampton, UK; 11 NIHR Southampton Respiratory Biomedical Research Unit, Southampton, UK

## Abstract

**Background:**

Childhood asthma is a common condition. Currently there is no validated objective test which can be used to guide asthma treatment in children. This study tests the hypothesis that the addition of fractional exhaled nitric oxide (F_E_NO) monitoring in addition to standard care reduces the number of exacerbations (or attacks) in children with asthma.

**Methods:**

This is a multi-centre, randomised controlled study. Children will be included of age 6–16 years who have a diagnosis of asthma, currently use inhaled corticosteroids (ICSs) and have had an exacerbation in the previous 12 months. Exclusion criteria include being unable to provide F_E_NO measurement at baseline assessment, having another chronic respiratory condition and being currently treated with maintenance oral steroids. Participants will be recruited in both primary and secondary care settings and will be randomised to either receive asthma treatment guided by F_E_NO plus symptoms (F_E_NO group) or asthma treatment guided by symptoms only (standard care group). Within the F_E_NO group, different treatment decisions will be made dependent on changes in F_E_NO. Participants will attend assessments 3, 6, 9 and 12 months post randomisation. The primary outcome is asthma exacerbation requiring prescription and/or use of an oral corticosteroid over 12 months as recorded by the participant/parent or in general practitioner records. Secondary outcomes include time to first attack, number of attacks, asthma control score and quality of life. Adherence to ICS treatment is objectively measured by an electronic logging device. Participants are invited to participate in a “phenotyping” assessment where skin prick reactivity and bronchodilator response are determined and a saliva sample is collected for DNA extraction. Qualitative interviews will be held with participants and research nurses. A health economic evaluation will take place.

**Discussion:**

This study will evaluate whether F_E_NO can provide an objective index to guide and stratify asthma treatment in children.

**Trial registration:**

ISRCTN, ISRCTN67875351. Registered on 12 April 2017. Prospectively registered.

## Administrative information

Note: the numbers in curly brackets in this protocol refer to SPIRIT checklist item numbers. The order of the items has been modified to group similar items (see http://www.equatornetwork.org/reporting-guidelines/spirit-2013-statementdefining-standard-protocol-items-for-clinical-trials/).

**Table:** 

Title {1}	Reducing Asthma Attacks in Children using Exhaled Nitric Oxide as a biomarker to inform treatment strategy - a randomised trial (RAACENO)
Trial registration {2a and 2b}	ISRCTN, ISRCTN67875351. Registered on 12 April 2017. Prospectively registered.
Protocol version {3}	Version 5; 08.03.19
Funding {4}	National Institute for Health Research (NIHR) Efficacy and Mechanism Evaluation (EME) programme, project number 15-18-14
Author details {5a}	^1^Child Health, University of Aberdeen, Aberdeen, UK. ^2^Centre for Healthcare Randomised Trials, University of Aberdeen, Aberdeen, UK. ^3^Medical Statistics Team, Institute of Applied Health Sciences, University of Aberdeen, Aberdeen, UK. ^4^Respiratory Sciences, University of Leicester, Leicester, UK. ^5^Department of Paediatric Respiratory Medicine and Allergology, Erasmus MC – Sophia Children’s Hospital, Rotterdam, Netherlands. ^6^Postgraduate Education Group, Institute of Applied Health Sciences, University of Aberdeen, Aberdeen, UK. ^7^Health Economics Research Unit, Institute of Applied Health Sciences, University of Aberdeen, Aberdeen, UK. ^8^Observational and Pragmatic Research Institute Pte Ltd, Singapore, Singapore. ^9^Centre of Academic Primary Care, University of Aberdeen, Aberdeen, UK. ^10^Primary Care and Population Sciences, University of Southampton, Southampton, UK. 11NIHR Southampton Respiratory Biomedical Research Unit, Southampton, UK.
name and contact information for the trial sponsor {5b}	**Co-sponsor 1.** University of Aberdeen, Foresterhill House Annexe, Foresterhill, Aberdeen, AB25 2ZB, researchgovernance@abdn.ac.uk
	**Co-sponsor 2.** NHS Grampian, Foresterhill House Annexe, Foresterhill, Aberdeen, AB25 2ZB, researchgovernance@abdn.ac.uk
Role of sponsor {5c}	The sponsor played no part in study design; and will play no part in the collection, management, analysis, and interpretation of data; writing of the report; and the decision to submit the report for publication.

## Introduction

### Background and rationale {6a}

There are one million children in the UK with asthma [1]. Although asthma cannot be cured, there is effective treatment to control symptoms and reduce the risk of asthma attacks. There is an urgent need to identify and validate a biomarker to guide asthma treatment and provide objective measurements to support clinical decision-making, e.g. when to use which treatment and when to step down treatment. Fractional exhaled nitric oxide (F_E_NO) is a surrogate marker for eosinophilic airway inflammation [2–5] and, since eosinophils are seen in the airways of people with asthma [6], it was assumed that F_E_NO measurements could be used to improve asthma control.

The evidence from clinical trials, however, is that the addition of F_E_NO monitoring to usual care does not improve asthma control [7, 8]. Sputum eosinophilia is known to be a temporary phenomenon in children [9], and this temporality at least partly explains the poor correlation between F_E_NO and current and future asthma control [10–13] and also the failure of F_E_NO-guided treatment to improve symptomatic asthma control [14]. In contrast, changes in F_E_NO concentrations are more clearly observed in the context of asthma attacks (sometimes just called attacks). For example, F_E_NO rises before an attack [15] and falls afterwards [16]. The relationship between F_E_NO and attack is replicated by the correlation between airway eosinophilia and asthma attack; asthma treatment guided by airway eosinophilia reduces asthma attacks in adults [17] and children [18] (the latter with borderline significance in a small study). Of note, asthma control was not improved in the intervention arm compared to the standard treatment arm in these studies [17, 18]. Eosinophilic inflammation is suppressed by treatment with inhaled corticosteroids (ICS), and F_E_NO increases after unsuccessful reduction [19] or cessation [20] of ICS. Together these observations show how airway eosinophilia is an index of attack risk (but not of poor symptomatic asthma control) which can be suppressed with ICS and which is correlated with F_E_NO.

Until recently, the application of F_E_NO into clinical practice has been uncertain, as the answer to the question “What is a significant change in F_E_NO?” was unknown. Previous trials adopted F_E_NO cut-offs based on comparisons between children with and without asthma or simply empirical values, e.g. 20, 30, 40 parts per billion (ppb). Our recent work has demonstrated that F_E_NO values may rise and fall, independently of asthma, by up to 50% over 2- and 4-month intervals [12]. Based on these observations, we will, for the first time in a clinical trial, use percentage change in F_E_NO to interpret repeated F_E_NO measurements.

We will deliver a rigorous and adequately powered trial to confirm whether F_E_NO-guided algorithm-based asthma treatment prevents asthma attacks. This trial is timely given the 2014 Diagnostic Guideline from the National Institute for Health and Care Excellence (NICE) [21] which stated that “F_E_NO measurement is recommended as an option to support asthma management…in people who are symptomatic despite using inhaled corticosteroids” and also stated that “The Committee … accepted there is a need for more evidence on which protocols offer the safest and most optimal asthma management when used in UK clinical practice”. This trial will evaluate the clinical efficacy of our algorithm-guided intervention on asthma attacks while describing the relationship between F_E_NO, asthma control and attacks. Our hypothesis is that the proportion of children with ≥ 1 asthma attack over 12 months will be reduced when asthma treatment guided by F_E_NO plus symptoms is compared to treatment guided only by symptoms.

### Aim and objectives {7}

The aim of the study is to compare treatment guided by F_E_NO and symptoms against treatment guided by symptoms alone (standard care), in children with asthma who are at risk of an asthma attack, in terms of the presence of any asthma attacks over 12 months requiring prescription and/or use of an oral corticosteroid (OCS).

The objectives are:

- To recruit 502 eligible children
- For recruited children to complete an assessment including spirometry, Asthma Control Test (ACT) or the Childhood Asthma Control Test (CACT) and F_E_NO at baseline
- To randomise children to intervention (treatment guided by F_E_NO and symptoms) or standard care (treatment guided by symptoms alone)
- To monitor adherence to inhaled corticosteroid treatment with an electronic logging device
- To repeat F_E_NO and ACT/CACT at 3, 6, 9 and 12 months and change asthma treatment according to the trial protocol
- On an optional basis, to collect saliva for DNA isolation to allow genetic analysis in a separate study
- On an optional basis, for children (approximately 200) to have skin prick reactivity and bronchodilator response determined for a mechanistic study
- To undertake a qualitative process evaluation of approximately 20 children and approximately 15 members of trial staff representing a number of roles across different sites, to explore experiences and acceptability of the intervention
- To undertake an economic evaluation to assess the healthcare costs (e.g. asthma-related hospital admissions and visits to/from relevant health professionals, asthma medications) and other related costs (e.g. parents’ time off work) and quality of life effects (quality-adjusted life years [QALYs]) of the intervention compared to routine care
- To compare the primary and secondary outcomes between treatment arms.

### Trial design {8}

This is a multi-centred randomised trial comparing the efficacy of asthma treatment guided by symptoms and F_E_NO with asthma treatment guided by symptoms alone for risk of asthma attack.

The research design also includes an evaluation of healthcare costs (including primary and secondary care contacts and asthma treatment). The qualitative process evaluation using established research techniques will explore experiences and determine the acceptability of the intervention by interviewing 20 children in the intervention arm and 15 research nurses until saturation of themes is achieved

## Methods: participants, interventions and outcomes

### Study setting {9}

We are recruiting children in secondary care sites across the UK and in primary care centres in the East of England.

### Eligibility criteria {10}

The inclusion criteria are:

1. Asthma diagnosed or confirmed by consultant paediatrician or respiratory/asthma specialist nurse (or Read code for asthma if recruited in primary care)
2. Patient aged 6 years or older and has not reached the date of 16th birthday (children < 6 years find it difficult to provide F_E_NO measurements [22])
3. Currently prescribed ICSs in a device that can be fitted with a Smartinhaler (electronic logging device): the maximum dose for children aged < 12 is 1000 μg budesonide equivalent (BUD) per day; the maximum dose for children aged ≥ 12 is 2000 μg BUD per day.
4. Parent/patient-reported asthma attack treated with at least one course of OCS in the 12 months prior to recruitment.

The exclusion criteria are:

1. Unable to provide F_E_NO measurement at baseline assessment
2. Other chronic respiratory conditions which also manifest attacks
3. Current treatment with maintenance oral steroids.

### Who will take informed consent? {26a}

Consent is taken by researchers trained in Good Clinical Practice (GCP) and with experience in working with children and young people. Consent to participate in the qualitative interviews is taken from qualitative researchers with GCP training. Written consent is obtained from parent(s)/carer(s) and (where appropriate) from the participant. If the child does not provide written consent, he/she will be asked to give verbal assent.

### Additional consent provisions for collection and use of participant data and biological specimens {26b}

Consent includes the option to give permission to collect saliva for DNA extraction and for linkage of data to other data sources.

### Interventions

#### Explanation for the choice of comparators {6b}

The comparator is F_E_NO. Justification for this is given in the “Background and rationale” section.

#### Intervention description {11a}

In the intervention arm, asthma treatment is guided by F_E_NO and symptoms. Table 1 describes the treatment steps. The experimental intervention and subsequent adjustment of treatment steps are applied at recruitment and at each of the follow-up visits (3, 6, 9 and 12 months). In the standard care arm, asthma treatment is guided by symptoms alone; adjustment of treatment steps are applied at recruitment and at each of the follow-up visits (3, 6, 9 and 12 months). Table 2 describes the treatment steps, which are in accordance with national guidelines [23].

**Table 1:** Treatment steps for the experimental intervention. More details in relation to the treatment steps are provided in Appendix 1 of the supplement; a detailed decision tree is given in Appendix 2 of the supplement

Step	Algorithm 1 (F_E_NO high)	Algorithm 2 (F_E_NO not high)
1	Short-acting beta-agonist (SABA) as required only	SABA as required only
2	Budesonide (or beclomethasone) 200 μg daily plus SABA	Budesonide (or beclomethasone 200 μg twice daily plus SABA
3	Budesonide (or beclomethasone) 400 μg OR fluticasone 200 μg daily plus SABA	Budesonide (or beclomethasone) 400 μg OR fluticasone 200 μg daily plus SABA
4	Budesonide (or beclomethasone) 800 μg OR fluticasone 500 μg daily plus SABA	Add long-acting beta-agonist (LABA)
5	*Only for* ≥ *12-year-olds:* Budesonide (or beclomethasone 1600 μg daily or fluticasone 1000 μg daily plus SABA. *Go to step 6 for < 12-year-olds*	Add leukotriene receptor antagonist
6	Add LABA in fixed dose combination	Budesonide 800 μg or fluticasone 500 μg daily in fixed dose combination
7	Add leukotriene receptor antagonist	*Only for* ≥ *12-year-olds.* Budesonide (or beclomethasone 1600 μg daily or fluticasone 1000 μg daily plus SABA*. Go to step 8 for < 12-year-olds*
8	Refer for specialist assessment	Refer for specialist assessment

**Table 2:** Treatment steps for the control intervention. More details in relation to the treatment steps are provided in Appendix 1; a detailed decision tree is shown in Appendix 2

Treatment step	Daily ICS dose μg budesonide or equivalent	Delivery device used prior to enrolment used after enrolment	
1	0	No ICS	Short-acting beta-agonist (SABA) as required only
2	200	Very low dose ICS	Budesonide (or equivalent) 100 μg twice daily plus SABA
3	400	Low dose ICS	Budesonide (or equivalent) 200 μg twice daily plus SABA
4	400	ICS + long-acting beta-agonist (LABA) combination inhaler	Budesonide (or equivalent) 200 μg twice daily plus SABA and LABA (dose depending on ICS molecule used)
5	400	Add on leukotriene receptor antagonist (LTRA)‡	Budesonide (or equivalent) 200 μg twice daily plus SABA, LABA and LTRA
6	800	High dose ICS	Budesonide (or equivalent) 400 μg twice daily plus SABA, LABA (dose depending on ICS molecule used) and LTRA
7 For 12–16 year olds (go to step 8 for children < 12)	1600	High dose ICS	Budesonide (or equivalent) 800 μg twice daily plus SABA, LABA and LTRA
8			Refer for specialist opinion

### Algorithm

Web-based software is used to apply a decision tree algorithm which is described in the supplement. At each assessment (baseline and 3, 6, 9 and 12 months) the researcher enters the participant F_E_NO, CACT or ACT score and the current medication. At the 3, 6, 9 and 12 months assessments, information on adherence to inhaled corticosteroid medication is also entered into the software. The algorithm within the web-based software considers the participant’s age, current medication, asthma control, adherence (at the 3, 6, 9 and 12 months assessments) and (in the F_E_NO-guided arm) F_E_NO before recommending what treatment should be taken. Within the F_E_NO-guided arm, there are two “ladders” of escalating and reducing treatment steps, and the change in F_E_NO determines which ladder is applied. For example, if the participant has poor asthma control and high F_E_NO then their ICS treatment is increased, whereas if their F_E_NO has not risen then they start long-acting beta-agonist (LABA) treatment. Based on our earlier work [12], a change of > 50% is defined as a significant change. The algorithm includes a number of “safety netting” instructions which allow only one treatment step up in the context of (1) elevated F_E_NO and controlled symptoms, (2) elevated F_E_NO, uncontrolled symptoms and poor adherence and (3) persistently low F_E_NO and uncontrolled symptoms. In both arms, the algorithm allows for a single step up in treatment when the participant has poorly controlled symptoms but has poor adherence, and also recommends “refer specialist opinion” (i.e. the researcher should ask an asthma specialist to review the participant) if the highest level of treatment is reached but control remains poor or one of the “safety netting” instances occurs. At their discretion, local clinical teams may choose not to apply the algorithm recommendations and to make their own clinical recommendation on treatment, and if they do this, the reason is recorded.

Table 3 summarises the information captured at each assessment. At each visit, the following is assessed:

- Asthma symptoms are measured using the ACT (or CACT [24]), and a score of < 20 is defined as poor control.
- F_E_NO, using the standard methodology. Although F_E_NO is measured in children in the standard care arm at recruitment (and at each of the follow-up visits), the results of the F_E_NO will not be used in treatment decisions for this arm of the trial. F_E_NO results are recorded once the child has left the room.
- Forced expiratory volume in one second (FEV_1_), using the standard methodology.
- Inhaler technique.

**Table 3:** Timing of outcomes to be assessed

	Time point				
	Baseline	3 months	6 months	9 months	12 months
F_E_NO	✓	✓	✓	✓	✓
Smartinhaler® data		✓	✓	✓	✓
Respiratory case report form (current medication, recent asthma history and attacks, inhaler technique, etc.)	✓				
Asthma Control Test	✓	✓	✓	✓	✓
Paediatric Asthma Quality of Life Questionnaire	✓				✓
Spirometry (FEV_1_) and height	✓	✓	✓	✓	✓
Weight	✓				✓
Asthma attacks	✓	✓	✓	✓	✓
Asthma-related healthcare and other related resource use	✓	✓	✓	✓	✓
Mechanistic studies					
Bronchodilator response (optional)	✓				
Skin prick testing (optional)	At any assessment				
Saliva for DNA extraction (optional)	At any assessment				

At the baseline and 12 months assessments, the following are ascertained:

- Weight
- Quality of life using the Paediatric Asthma Quality of Life Questionnaire (PAQLQ) [25].

At the 3, 6, 9 and 12 months assessments, the following are assessed:

- Adherence to treatment
- Asthma exacerbation since the last study visit
- Healthcare resource use since the last study visit.

Additionally, participants have the option of providing a saliva sample for DNA extraction and analysis, having skin prick reactivity to egg, cat dander, grass and house dust mite and bronchodilator responsiveness assessed. Methodologies are described in the supplement.

### Adherence to inhaler corticosteroid treatment

Adherence is determined by the researcher at all visits. The definition of adequate adherence is either > 70% adherence as measured by the Smartinhaler® electronic logging device or by participant/parent report of being adherent most or all of the time. This definition allows for missing Smartinhaler® data, e.g. at baseline assessment, failure of the device or non-availability of the device, and also the real-world scenario where there is a discrepancy between the Smartinhaler® data and participant/parent report.

### Qualitative interviews

In a qualitative process evaluation to explore experiences and ascertain acceptability of the intervention, and to solicit in-depth feedback on the process of taking part in this trial, children in the intervention arm (*n* = 20) will be invited to give a qualitative interview with an experienced qualitative researcher. A range of trial staff representing a number of roles and across different sites (*n* = 15) will also be interviewed to understand the feasibility of intervention delivery from provider perspectives and to access any additional observations made around acceptability/process. We will interview staff in Aberdeen initially and then researchers in Scotland (due to proximity to the trial office, where the qualitative researcher will be based) and English centres selected for success in participant recruitment and retention in the trial. Interviews may be carried out over the telephone. Research nurses will be invited for interview who do and who do not have previous expertise in paediatric respiratory medicine, and also research nurses who have more than approximately 10 years’ experience and approximately less than five years’ experience to gain insight into whether respiratory and/or research experience affect perspectives. Our interviews will explore with healthcare staff decision-making around not applying the algorithm: for example, does confidence in the algorithm grow over time? Is there “intelligent non-concordance”, e.g. reluctance to increase (or step up) treatment if the participant is already on high level treatment or also reluctance to not step down if on low treatment (especially if stopping)? Interviews will continue until saturation of emerging themes. Further details are in the supplement.

### Criteria for discontinuing or modifying allocated interventions {11b}

Adherence to intervention is facilitated by the web-based design of the treatment algorithm.

### Strategies to improve adherence to intervention {11c}

Adherence to the intervention is facilitated by the web-based software which presents the researcher clear instructions as to what change in treatment (if any) is required.

### Relevant concomitant care permitted or prohibited during the trial {11d}

Usual care for participants continues throughout the trial. There is nothing prohibited.

### Provisions for post-trial care {30}

Standard care is provided within the UK National Health Service (NHS).

### Outcomes {12}

#### Primary outcome

The primary outcome is prescription for (and/or use of) ≥ 1 course of OCS for asthma attacks in the 12 months after randomisation (yes/no). The decision to prescribe OCS is made by clinicians independent of the research team and working in accordance with the national guidelines [23]. The primary outcome is captured from parental report at the 3, 6, 9 and 12 month assessments. Where data are not available at 12 months, the general practice (GP) at which the participant is registered is contacted to capture primary outcome data.

#### Secondary outcomes

- Time to first attack
- Number of attacks during follow-up
- Need for unscheduled healthcare assessment during follow-up (yes/no)
- Number of unscheduled health assessments
- Asthma control during follow-up (i.e. age-appropriate ACT score ≥ 20 [24])
- Spirometry during the 12 months follow-up (i.e. %FEV_1_, standardised to Global Lung Function Initiative [26])
- F_E_NO during the 12 months follow-up
- Dose of ICS during the 12 months follow-up (i.e. daily dose of budesonide equivalent averaged over 3 months)
- Paediatric Asthma Quality of Life Questionnaire (PAQLQ) [25] score at 12 months
- Qualitative outcomes from interviews
- Health economic evaluation (derived from GP records and participant reported data).

### Participant time line {13}

**Fig. 1:**
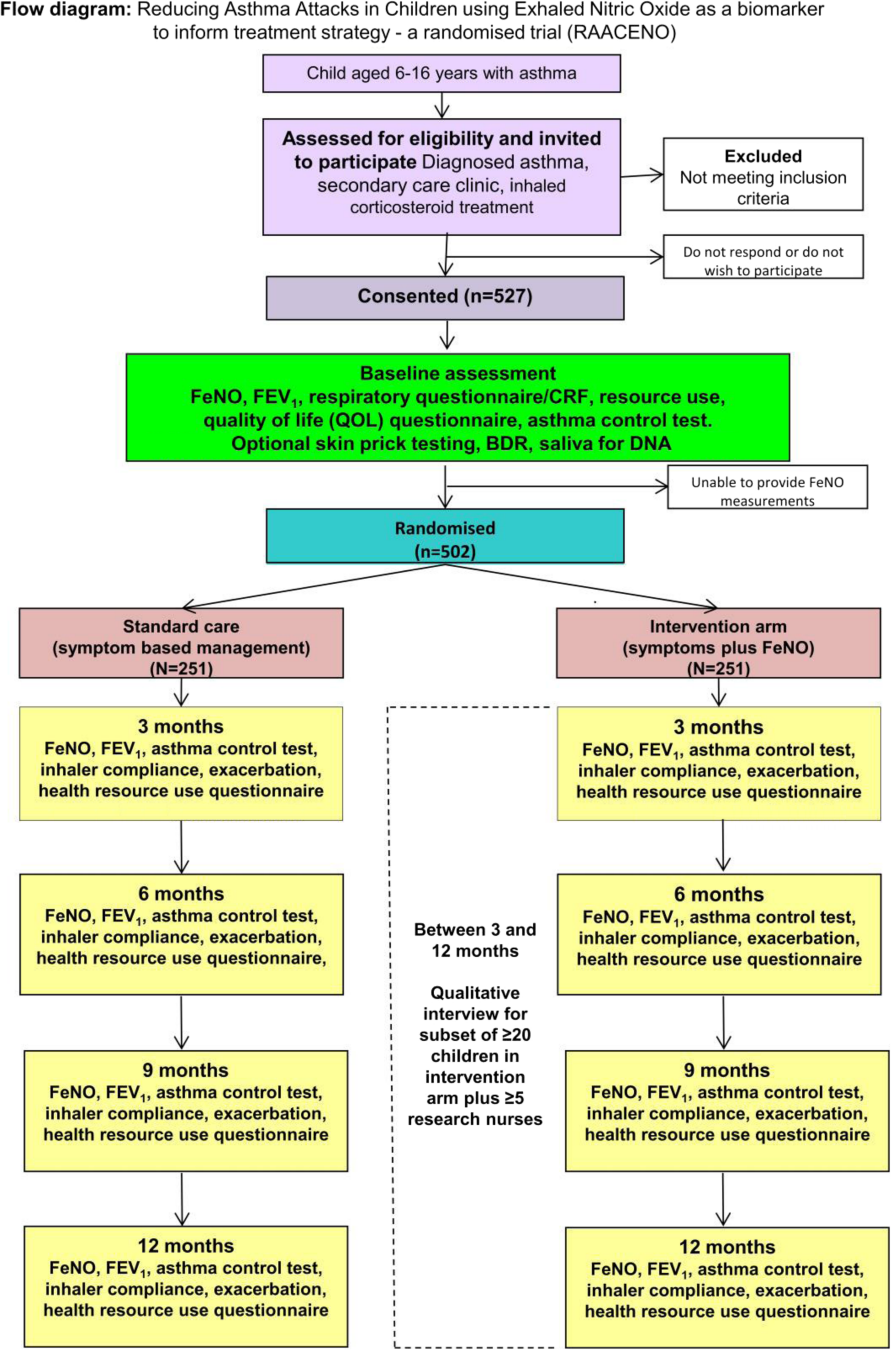
Flow diagram illustrating the participant’s journey through the RAACENO trial

See Fig. 1 for the participant’s time line through the trial.

### Sample size {14}

Our meta-analysis finds a relative 33% reduction in the proportion with ≥ 1 attack receiving F_E_NO-guided treatment [27]. Assuming an attack proportion of 44% for the symptom-guided treatment group and 29.5% for the intervention group, we have 90% power with 5% significance (two-sided) if we recruit 238 children per group. Allowing for 5% incomplete follow-up, we will recruit 502 children (i.e. 251 per group).

### Recruitment {15}

Eligible individuals are identified by their usual clinical team and are sent a letter of invitation which is accompanied by a short participant and parent information sheet. Parent information sheet and the age-appropriate children’s information sheet. A follow-up telephone call is arranged to establish whether an appointment should be made for a face-to-face meeting where eligibility is confirmed, consent taken, baseline details collected and randomisation performed.

### Assignment of interventions: allocation

#### Sequence generation {16a}

After consent is given, participants are randomly allocated to either the intervention or standard care group using a minimisation algorithm, with stratification by recruiting centre, age (< 11 or ≥ 11 years) and asthma severity (British Thoracic Society/ Scottish Intercollegiate Guidelines Network [BTS/SIGN] treatment step 2, 3 or 4), including a random element (20%). The primary care centres are collectively considered as one recruiting centre for randomisation.

#### Concealment mechanism {16b}

The web-based randomisation system ensures allocation concealment.

#### Implementation {16c}

The allocation sequence generation is embedded in the trial web site. Research nurses based at sites enroll participants and randomise them using the web-based randomisation system.

### Assignment of interventions: blinding

#### Who will be blinded {17a}

There can be no blinding of participants, parents, clinical staff or the central trial team to the allocated trial arm. Participants in the standard care arm are blinded to their F_E_No results until they have completed the 12 months follow-up.

#### Procedure for unblinding if needed {17b}

There is no requirement for emergency unblinding procedures.

### Data collection and management

#### Plans for assessment and collection of outcomes {18a}

Data are collected at baseline and at 3, 6, 9 and 12 months.

#### Plans to promote participant retention and complete follow-up {18b}

There is a 6-week visit window around each of the follow-up appointments. Where possible, study follow-up visits are timed to coincide with routine clinic appointments. There are no additional plans to enhance retention to RAACENO.

### Data management {19}

All data are entered by site staff onto a web-based case report form. Data are held on a secure server at the University of Aberdeen. The central trials team monitor data entry and ensure that missing data are addressed as soon as possible after detection.

### Confidentiality {27}

Data are stored in accordance with GCP and with the UK Data Protection Acts 1998 and 2018.

### Plans for collection, laboratory evaluation and storage of biological specimens for genetic or molecular analysis {33}

Saliva samples are collected in Oragene collection kits (DNA Genotek, Ottawa, ON, Canada) for later DNA extraction and analysis. A candidate gene is rs1042713.

### Analysis

#### Statistical methods for primary and secondary outcomes {20a}

Analysis will be by intention-to-treat. To determine whether the intervention leads to reduction in the primary outcome, logistic regression will be used to compare the primary outcome (yes/no) between treatment groups adjusting for relevant baseline factors known to be strongly related to attack at 12 months (age, gender, the age mother left full-time education, asthma severity and centre). Number of attacks will be analysed using Poisson regression adjusting for the same baseline factors. Time to first attack will be compared between groups using Cox regression. Secondary outcomes including ACT, F_E_NO, FEV_1_ and dose of ICS will be compared between treatment groups using linear mixed effects models to account for the correlation between repeated measures. The benefit of this approach is inclusion of all individuals where there is ≥ 1 clinical assessment. Unscheduled healthcare attendance (yes or no) will be compared between treatment groups using generalised estimating equations and, if deemed appropriate, the number of unscheduled healthcare attendances will be compared using Poisson regression. Full details will be described in the statistical analysis plan for the study. Comparison of quality of life (using the PAQLQ) at the final assessment (12 months) between treatment groups will be assessed using analysis of covariance, adjusting for minimisation variables, baseline values and other appropriate baseline predictors. The influence of any missing data on the robustness of the findings will be examined using sensitivity analyses incorporating multiple imputation or other relevant strategies under alternative assumptions.

#### Interim analyses {21b}

There will be no interim analyses.

#### Methods for additional analyses (e.g. subgroup analyses) {20b}

We will explore whether outcomes are different between groups for the stratification variables (gender, age group, asthma severity). Additionally we will explore whether an attack was precipitated by an upper respiratory tract infection, whether the participant was treated with a leukotriene receptor antagonist or whether the participant was skin prick positive. The analytical framework for the health economic evaluation will adopt both a cost-effectiveness approach, assessing health gains in terms of asthma exacerbations prevented, and a cost-utility approach, assessing gains in QALYs. A thematic approach will be used to analyse qualitative data.

#### Methods in analysis to handle protocol non-adherence and any statistical methods to handle missing data {20c}

Analysis will be by intention-to-treat. We do not plan to impute missing values, but may consider use of multiple imputation or other strategies within the sensitivity analysis (see the section “Statistical methods for primary and secondary outcomes”).

#### Plans to give access to the full protocol, participant-level data and statistical code {31c}

The full protocol is available as a supplement. Non-identifiable participant-level data may be available on request to the Chief Investigator (CI), Professor Turner (s.w.turner@abdn.ac.uk).

### Oversight and monitoring

#### Composition of the coordinating centre and Trial Steering Committee {5d}

The immediate trial team based in the coordinating centre (CI, trial manager, data coordinator) meets weekly. On a monthly basis, the immediate team is joined by the wider team based in the coordinating centre (statistician, health economist, qualitative researcher). A Project Management Group (PMG) and Trial Steering Committee (TSC) oversee the project. The PMG meets every 3 months and comprises the CI, grant holders (including clinical, methodological, statistical, health economic and qualitative expertise) and the trial office staff. The TSC meets every 6 months and includes an independent chair, clinical and methodological expertise and lay representative.

#### Composition of the Data Monitoring Committee, its role and reporting structure {21a}

The Data Monitoring Committee meets every 6 months. It includes an independent chair and independent members with clinical and methodological expertise, and it reports to the chair of the TSC.

#### Adverse event reporting and harms {22}

Within RAACENO, we only record any adverse events (AEs) and serious adverse events (SAEs) relating to use of the NIOX VERO device or other study assessments. All AEs (including SAEs) meeting the criteria for recording within RAACENO are recorded from the time a participant consents to join the trial until the last trial visit. The Investigator asks about the occurrence of AEs at every visit. Open-ended and non-leading verbal questioning of the participant is used to enquire about AE occurrence. The Investigator (or delegate) reviews appropriate documentation (e.g. hospital notes, laboratory and diagnostic reports) related to the event. The Investigator (or delegate) records all relevant information on the AE form. Site staff are responsible for notifying the trial office of any AEs. The CI or delegate will report any related and unexpected serious AEs to the Research Ethics Committee (REC) within 15 days of the CI becoming aware of it. All related serious AEs are summarised and reported to the REC, the funder and the TSC in their regular progress reports. An asthma attack (defined as an increase in asthma symptoms requiring treatment with oral corticosteroids) is the primary outcome and is not an AE.

#### Frequency and plans for auditing trial conduct {23}

The trial office monitors aspects of the study on an ongoing basis as described in the study monitoring plan. The trial is monitored and audited by the sponsor. Individual sites may be monitored by their local Research and Development (R&D) departments.

#### Plans for communicating important protocol amendments to relevant parties (e.g. trial participants, ethical committees) {25}

Changes to the protocol require the trial office to seek permission from the funder, sponsor, REC and NHS R&D offices.

#### Dissemination plans {31a}

We will develop a publication and dissemination plan to include conference presentation(s) and journal publication(s). We plan to write to all participants and their families to inform them of the trial results. We will also plan dissemination to relevant patient and clinical interest groups.

## Discussion

Childhood asthma is a common condition, and there is a need for an objective test to help guide asthma management [28]. Additionally there is a desire to recognise the heterogeneity of asthma by moving away from a “one size fits all” management strategy, and instead stratify treatment to the individual [29]. The RAACENO study will rigorously evaluate whether treatment guided by symptoms plus F_E_NO reduces asthma attacks compared to symptom-only treatment.

There have been eight previous randomised controlled trials (RCTs) which have used F_E_NO to guide asthma treatment. In only one of these was there an improvement in asthma control, and in four there were reduced asthma attacks. This present study differs from previous trials in (1) being powered on asthma attacks and not asthma control, (2) individualising change in F_E_NO by using percentage change and therefore not using the same cut-off values for the whole population and (3) having different treatment pathways within the F_E_NO-guided treatment arm depending on F_E_NO values.

A recent study where data from seven of the previous RCTs were pooled has found that a relatively large change in F_E_NO occurred before asthma status changed [30]. This result is consistent with that of a previous study [12], and together these data support the relatively large change in F_E_NO used to trigger change in treatment in the RAACENO study. This recent work also supports the RAACENO methodology by finding that percent change in F_E_NO, and not absolute change in F_E_NO, preceded a change in asthma status [30]. However, the paper by Fielding et al. [30] observed that changes in percentage of F_E_NO preceded loss of asthma control but not of asthma attack, but in contrast RAACENO is powered on attacks and not control. The methodology used in RAACENO is substantially different from those of the previous RCTs. The RAACENO trial will report on its findings in early 2021.

### Trial status

Recruitment was completed on 8th August 2019. The current protocol is version 5 (dated 08/03/2019).

## Data Availability

Data may be available for collaborators on request to the CI, Professor Turner (s.w.turner@abdn.ac.uk).

## Abbreviations

ACT: Asthma Control Test
AE: Adverse event
BTS/SIGN: British Thoracic Society/Scottish Intercollegiate Guidelines Network
BUD: Budesonide equivalent
CACT: Childhood Asthma Control Test
CHaRT: Centre for Healthcare Randomised Trials
CI: Chief Investigator
DMC: Data Monitoring Committee
DNA: Deoxyribonucleic acid
EME: Efficacy and Mechanism Evaluation
F_E_NO: Fractional exhaled nitric oxide
FEV_1_: Forced expiratory volume in one second
GCP: Good Clinical Practice
GP: General practitioner, general practice
ICS: Inhaled corticosteroid
ISRCTN: International Standard Randomised Controlled Trial Number
NHS: National Health Service
NICE: National Institute for Health and Care Excellence
OCS: Oral corticosteroid
PAQLQ: Paediatric Asthma Quality of Life Questionnaire
PMG: Project Management Group
ppb: Parts per billion
QALY: Quality-adjusted life year
R&D: Research and Development
RAACENO: Can we Reduce Asthma Attacks in Children using Exhaled Nitric Oxide
RCT: Randomised controlled trial
REC: Research Ethics Committee
SAE: Serious adverse event
TSC: Trial Steering Committee
UK: United Kingdom

## References

[1] Asthma UK (2017). Asthma facts and FAQs.

[2] Piacentini GL, Bodini A, Costella S, Vicentini L, Mazzi P, Sperandio S, Boner AL (1999). Exhaled nitric oxide and sputum eosinophil markers of inflammation in asthmatic children. Eur Respir J.

[3] Warke TJ, Fitch PS, Brown V, Taylor R, Lyons JD, Ennis M, Shields MD (2002). Exhaled nitric oxide correlates with airway eosinophils in childhood asthma. Thorax.

[4] Pontin J, Blaylock MG, Walsh GM, Turner SW (2008). Sputum eosinophil apoptotic rate is positively correlated to exhaled nitric oxide in children. Pediatr Pulmonol.

[5] Payne DN, Adcock IM, Wilson NM, Oates T, Scallan M, Bush A (2001). Relationship between exhaled nitric oxide and mucosal eosinophilic inflammation in children with difficult asthma, after treatment with oral prednisolone. Am J Respir Crit Care Med.

[6] Bousquet J, Chanez P, Lacoste JY (1990). Eosinophilic inflammation in asthma. N Engl J Med.

[7] Petsky HL, Kew KM, Chang AB (2016). Exhaled nitric oxide levels to guide treatment for children with asthma. Cochrane Database Syst Rev.

[8] Lu M, Wu B, Che D, Qiao R, Gu H (2015). FeNO and asthma treatment in children: a systematic review and meta-analysis. Medicine.

[9] Fleming L, Tsartsali L, Wilson N, Regamey N, Bush A (2012). Sputum inflammatory phenotypes are not stable in children with asthma. Thorax.

[10] Roberts G, Hurley C, Bush A, Lack G (2004). Longitudinal study of grass pollen exposure, symptoms, and exhaled nitric oxide in childhood seasonal allergic asthma. Thorax.

[11] Piacentini GL, Peroni DG, Bodini A (2009). Childhood Asthma Control Test and airway inflammation evaluation in asthmatic children. Allergy.

[12] Cutts R, Turner S (2013). Longitudinal measurements of exhaled nitric oxide in children—what is a significant change in FE(NO)?. Pediatr Allergy Immunol.

[13] Waibel V, Ulmer H, Horak E (2012). Assessing asthma control: symptom scores, GINA levels of asthma control, lung function, and exhaled nitric oxide. Pediatr Pulmonol.

[14] Petsky HL, Cates CJ, Lasserson TJ, Li AM, Turner C, Kynaston JA, Chang AB (2012). A systematic review and meta-analysis: tailoring asthma treatment on eosinophilic markers (exhaled nitric oxide or sputum eosinophils). Thorax.

[15] Vahlkvist S, Sinding M, Skamstrup K, Bisgaard H (2006). Daily home measurements of exhaled nitric oxide in asthmatic children during natural birch pollen exposure. J Allergy Clin Immunol.

[16] Baraldi E, Azzolin NM, Zanconato S, Dario C, Zacchello F (1997). Corticosteroids decrease exhaled nitric oxide in children with acute asthma. J Pediatr.

[17] Green RH, Brightling CE, McKenna S (2002). Asthma exacerbations and sputum eosinophil counts: a randomised controlled trial. Lancet.

[18] Fleming L, Bush A (2012). Use of sputum eosinophil counts to guide management in children with severe asthma. Thorax.

[19] Zacharasiewicz A, Wilson N, Lex C (2005). Clinical use of noninvasive measurements of airway inflammation in steroid reduction in children. Am J Respir Crit Care Med.

[20] Pijnenburg MW, Hofhuis W, Hop WC, De Jongste JC (2005). Exhaled nitric oxide predicts asthma relapse in children with clinical asthma remission. Thorax.

[21] National Institute for Clinical Excellence (2014). Measuring fractional exhaled nitric oxide concentration in asthma: NIOX MINO, NIOX VERO and NObreath.

[22] Napier E, Turner SW (2005). Methodological issues related to exhaled nitric oxide measurement in children aged four to six years. Pediatr Pulmonol.

[23] British Thoracic Society and Scottish Intercollegiate Guidelines Network (2016). British guideline on the management of asthma.

[24] Liu AH, Zeiger R, Sorkness C (2007). Development and cross-sectional validation of the Childhood Asthma Control Test. J Allergy Clin Immunol.

[25] Juniper EF, Guyatt GH, Feeny DH, Ferrie PJ, Griffith LE, Townsend M (1996). Measuring quality of life in children with asthma. Qual Life Res.

[26] Quanjer PH, Stanojevic S, Cole TJ (2012). Multi-ethnic reference values for spirometry for the 3-95-yr age range: the global lung function 2012 equations. Eur Respir J.

[27] Turner S (2015). Exhaled nitric oxide and the management of childhood asthma – yet another promising biomarker “has been” or a misunderstood gem. Paediatr Respir Rev.

[28] Pijnenburg MW, Baraldi E, Brand PLP (2015). Monitoring asthma in children. Eur Respir J.

[29] Pavord ID, Beasley R, Agusti A (2018). After asthma: redefining airways diseases. Lancet.

[30] Fielding S, Pijnenburg M, de Jongste JC (2019). Change in FEV1 and FENO Measurements as predictors of future asthma outcomes in children. Chest.

